# Oaks and Climate Change: Contrasting Range Responses of Mediterranean and Temperate *Quercus* Species in the Western Palearctic

**DOI:** 10.1002/ece3.73055

**Published:** 2026-02-06

**Authors:** Elif Deniz Ülker, Çağatay Tavşanoğlu

**Affiliations:** ^1^ Institute of Science Hacettepe University Ankara Türkiye; ^2^ Division of Ecology, Department of Biology Hacettepe University Ankara Türkiye

**Keywords:** climate change, distribution range, Mediterranean oaks, *Quercus*, temperate oaks, western Palearctic region

## Abstract

Over the Quaternary, the geographic distributions of many species have experienced shifts in response to climatic changes. We examined the range‐shift patterns of six oak (*Quercus*) species occupying different climatic zones of the western Palearctic under both past and future climate conditions. Using ecological niche models, we reconstructed distributions during the Last Glacial Maximum (LGM, ~22,000 years before present), compared them to the Present, and projected future changes under two scenarios for 2081‐2100 (SSP1‐2.6 and SSP5‐8.5). Quantitative metrics of latitudinal centroid movement, range limits, and area change revealed consistent contrasts among climatic groups. During the LGM, temperate ( *Q. robur* and *Q. petraea* ) and transition‐zone ( *Q. cerris* and *Q*. *pubescens*) species contracted strongly, persisting in southern refugia across Anatolia, the Balkans, and the western Mediterranean, whereas Mediterranean oaks (*Q*. *coccifera* and *Q. suber* ) retained more stable ranges. Future projections suggest that temperate and transition‐zone species will undergo substantial range loss and poleward shifts, particularly under the pessimistic scenario, whereas Mediterranean oaks will experience limited latitudinal shifts but pronounced expansion in to northern latitudes and temperate regions. These findings indicate Mediterranean oaks are ecologically distinct from temperate and transition‐zone species, which show similar climate sensitivities. Our results emphasize the need for climate‐zone‐specific conservation strategies, including enhancing connectivity and genetic diversity for temperate and transition‐zone species, and prioritizing drought‐resilient populations and adaptive management for Mediterranean species, to support the long‐term resilience of European oak forests under ongoing and future climate change.

## Graphical abstract

We analyzed past and future range dynamics of six oak (*Quercus*) species across the western Palearctic using ecological niche models, reconstructing distributions for the Last Glacial Maximum, the present, and two future scenarios. Quantitative metrics of centroid shifts, range limits, and area change revealed that temperate and transition‐zone species experienced strong contractions and are projected to lose further habitat, whereas Mediterranean oaks retained stable historical ranges and are likely to expand northward. These findings highlight the ecological distinctness of Mediterranean oaks and emphasize the need for climate‐zone‐specific conservation strategies to enhance the resilience of European oak forests under climate change.

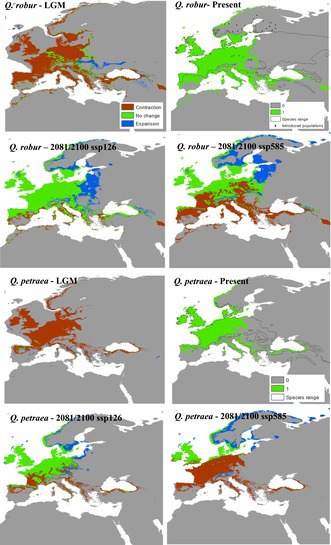

## 1 Introduction

The Quaternary period, beginning approximately 2.5 million years ago, involves frequent climatic changes. Given the extensive geomorphological and climatological records available, the Late Quaternary has emerged as a crucial period for understanding the evolutionary dynamics of contemporary communities and taxa (Hewitt 1996; Taberlet et al. 1998; Hewitt 1999; Petit et al. 2003; Svenning and Skov 2007; Waltari and Guralnick 2009; Médail and Diadema 2009). The effects of these global climatic fluctuations varied across continents, depending on several factors, such as distance from the ocean, ocean currents, continental mass, and regional topography. European forests experienced major transformations due to these alternating climate conditions during the Quaternary (Taberlet et al. 1998; Brewer et al. 2002; Petit et al. 2002). As the climate cooled and dried, glaciers expanded from the poles toward the south, especially during the Last Glacial Maximum (LGM), fragmenting the distribution of temperate and broad‐leaved forests in the western Palearctic and driving their contraction toward climatically suitable refugia, such as coastal zones, mountainous areas, and the southern parts of the region (Taberlet et al. 1998; Hewitt 1999; Svenning and Skov 2007; Schmitt and Varga 2012). Following glacial retreats, many species recolonized previously glaciated areas from these refugia, resulting in a range expansion that exemplifies the expansion–contraction model of Pleistocene biogeography (Provan and Bennett 2008; Ülker et al. 2018). Today, the pace of anthropogenic climate change is accelerating, and recent projections indicate that temperatures in Europe and parts of the Mediterranean are expected to rise by approximately 2°C–5°C by the year 2100 (Christensen et al. 2007; Pörtner et al. 2022). In addition to climatic drivers, human land use has also played a significant role in shaping the postglacial distribution of oaks across Europe. Since the early Holocene, agricultural expansion, silvicultural practices, and landscape modification have influenced oak dispersal and persistence, both by facilitating spread through habitat opening and by constraining populations through fragmentation and intensive land use (Svenning and Skov 2007). In particular, the long history of coppicing, wood pasture, and forest management has altered competitive dynamics and regeneration patterns of oak species (Petit et al. 2002). These direct and indirect human influences interact with climatic factors, contributing to the present‐day distribution patterns observed in many European oak species (Turner et al. 2008).

Ongoing global warming is already altering the functional traits, phenology, biotic interactions, and geographic distributions of species (Parmesan and Yohe 2003; Cavender‐Bares et al. 2004). Due to their long lifespans, tree species—and thus forest ecosystems—are particularly vulnerable to climate change, as their capacity to adapt to rapid environmental shifts is limited, although broad ecological amplitude and phenotypic plasticity may provide short‐term resilience, and in some groups, such as oaks (*Quercus*), extensive hybridization can increase the availability of genotypes suited to novel climates, which may have facilitated their ability to track climatic warming in the past (Kremer 2010). In the western Palearctic, climate change is having marked effects on both Mediterranean and temperate forest ecosystems (de Resco Dios et al. 2007; Nunes et al. 2021). Mediterranean forests face heightened risks of degradation and biodiversity loss due to the combined effects of rising temperatures, increased water scarcity, and altered precipitation patterns. These stressors contribute to habitat loss, range shifts, elevated fire and drought risk, ecological disruption, and the proliferation of pests and invasive species (Lindner et al. 2010). In temperate‐zone forests, including the diverse deciduous habitats of the western Palearctic, seasonal phenology is shifting primarily in response to warming, yet these shifts are also constrained by reduced water availability associated with declining annual precipitation (Maracchi et al. 2005).

Temperate deciduous broadleaf and mixed forests represent the largest terrestrial biome in the western Palearctic region, with the genus *Quercus* (oaks) serving as one of the dominant tree groups in these ecosystems (San‐Miguel‐Ayanz et al. 2016). Oaks are notable for their broad distribution and taxonomic diversity, comprising approximately 430 species across the Northern Hemisphere, including North America, Europe, Asia, and parts of North Africa (Carrero et al. 2020). Although Europe hosts only 28 oak species, *Quercus* is the most dominant tree genus on the continent (Carrero et al. 2020). The evolutionary history of *Quercus* dates back to the Eocene (~55 million years ago), when the genus underwent rapid diversification, likely driven by climatic shifts and the emergence of new habitats (Hipp et al. 2020). Over geological time, oaks expanded into various regions and adapted to a wide range of ecological niches, with continental drift and tectonic events further shaping their current geographical distribution. Glacial–interglacial cycles during the Pleistocene also influenced oak migration and diversification, leading to the emergence of localized species and subspecies. As such, climate change has been a major evolutionary driver in shaping the composition and distribution of the European oak flora (Petit et al. 2002; Hipp et al. 2020).

Due to their wide distribution, presence of subspecies, fragmented population structures, hybridization capacity, and occurrence across diverse climatic zones, the genus *Quercus* is of particular interest in ecology and evolutionary biology, offering valuable opportunities to address key biogeographical questions. In this context, our study investigates the climate‐based ecological niches of six *Quercus* species ( *Q. cerris* , *Q*. *coccifera*, *Q. petraea* , *Q*. *pubescens*, *Q. robur* , and *Q. suber* ) occurring in the western Palearctic region, representing Mediterranean and temperate climates, along with a third “transition zone” group comprising species whose distributions span both climatic zones. Using an ecological niche modeling (ENM) approach, we aimed to identify the suitable distribution areas and potential refugia for these species during the LGM. Additionally, we projected their potential distribution under future climate change scenarios. In selecting these six *Quercus* species, we aimed to represent the dominant oak taxa from distinct climatic zones of the western Palearctic: two species each from Mediterranean ( *Q. suber* and *Q*. *coccifera*), temperate ( *Q. robur* and *Q. petraea* ), and “transition” zones ( *Q. cerris* and *Q*. *pubescens*). This grouping reflects both ecological and phylogenetic diversity across *Quercus* sections (e.g., *Ilex*, *Cerris*, and *Quercus*). Although several previous studies have employed ENMs to examine the distribution oak species (e.g., Svenning et al. 2008; Vessella et al. 2017; López‐Tirado et al. 2018; Ülker et al. 2018; Suicmez and Avci 2023), few have explicitly compared niche responses across climatic zones and *Quercus* sections by using a uniform modeling framework. Our approach builds on and extends these studies by directly contrasting past and future range shifts among representative species from different biogeographic domains. Given the known differences in life history traits between Mediterranean and temperate oaks, we hypothesized that their climatic niches differ, resulting in distinct distributional responses to climate change across periods, including the LGM and the future. We also expected that the locations of LGM refugia would differ between Mediterranean and temperate oaks. Furthermore, we anticipated that transition‐zone oaks would exhibit intermediate responses, reflecting their position between the two climatic extremes.

## 2 Methods

### 2.1 Study Area and Species

The western Palearctic Region (12° W–52° E and 20°–72° N) features vast plains extending west‐northward into Russia, along with major east–west mountain ranges in the south, including the Alps, Pyrenees, and Carpathians (Hewitt 1996). Further south lies the Mediterranean basin where complex land‐sea interactions and a long history of human influence have shaped diverse ecosystems. Of particular relevance, Anatolia, located in the Asian portion of this region, is characterized by pronounced topographic and climatic heterogeneity (Şekercioğlu et al. 2011). The study region includes four major Köppen–Geiger climate types: cold (D) as the dominant type, followed by arid (B), temperate (C), and polar (E) climates (Peel et al. 2007).

The western Palearctic hosts 28 *Quercus* (oak) species, along with several subspecies and hybrids, representing the lowest oak species richness among major oak regions of the northern Hemisphere. These species are classified into four sections: *Quercus*, *Cerris*, *Ilex*, and *Ponticae*. For this study, we grouped species based on their primary distribution across climatic zones; however, some species span multiple zones, prompting us to define a third category, the “transition‐zone” group. We selected two representative species from each zone: temperate (*Q. robur and Q. petraea*), Mediterranean (*Q. coccifera and Q. suber*), and transition‐zone (*Q. cerris and Q. pubescens*). We followed Denk et al. (2017) for the species nomenclature and taxonomic classification.

### 2.2 Occurrence and Climate Data

Occurrence data were primarily compiled from the Global Biodiversity Information Facility (GBIF, https://www.gbif.org/), regional floras (Hedge and Yaltırık 1982; Tutin et al. 1964–1980; Vila‐Viçosa et al. 2023), and the European Forest Genetic Resources Program (EUFORGEN) (https://www.euforgen.org/species/). We applied a quality‐control process to all records, irrespective of their source or record type (“preserved specimen,” “observation,” “human observation”), to minimize spatial errors and taxonomic uncertainty. Specifically, after downloading data from GBIF (Table S1), we excluded records during data screening if: (i) coordinates were obviously erroneous (e.g., located in oceans or far outside the known native range), (ii) coordinates corresponded to the herbarium institution rather than the actual collection locality, or (iii) the location was inconsistent with well‐documented species distributions. This filtering approach ensured that only georeferenced records with plausible locations and confirmed species identities were retained for analysis. Despite the application of quality‐control filters and spatial thinning, uneven sampling effort across the western Palearctic may still introduce bias into occurrence data. Well‐surveyed regions such as central and western Europe are often overrepresented in biodiversity databases, whereas eastern Europe, parts of Anatolia, and North Africa remain comparatively under‐sampled, potentially increasing uncertainty in peripheral or poorly known areas. Although spatial thinning reduces clustering effects, it cannot fully compensate for gaps in geographic coverage, highlighting the need for targeted sampling and complementary sensitivity analyses in future studies.

For climatic data, we obtained 19 bioclimatic variables for each time period from WorldClim version 2.1 (Fick and Hijmans 2017), at a spatial resolution of 2.5 arc‐min (~4.63 km at the equator) (Table S2). Future climate projections (2081–2100) were based on three general circulation models (BCC‐CSM2‐MR, CNRM‐CM6‐1, and MIROC6) and two shared socioeconomic pathways: an optimistic scenario (SSP1‐2.6) and a pessimistic scenario (SSP5‐8.5). These scenarios project mean annual temperature increases of 0.2°C–1.8°C and 2.6°C–4.8°C, and atmospheric CO_2_ concentrations of approximately 450 ppm and 1350 ppm by 2100, respectively (Pörtner et al. 2022). For LGM projections, we used three paleoclimatic models: CCSM, MPI, and MIROC.

### 2.3 Ecological Niche Modeling

We employed an ENM approach to predict the potential impacts of climate change on the distribution patterns of the studied *Quercus* species across different time periods and climate scenarios. Specifically, we modeled species distributions under past climatic conditions during LGM (~22,000 years before present), under present conditions, and under two future scenarios for 2081‐2100 (SSP1‐2.6 and SSP5‐8.5).

During the data preparation stage, *SDMToolbox* (Brown 2014) was used in ArcGIS 10.6.1. (ESRI 2010) to remove the duplicate occurrence records and reduce spatial autocorrelation by applying spatial thinning. Nonetheless, some spatial bias may still persist, especially in under‐sampled regions, and could influence model predictions by underrepresenting suitable habitats in those areas. This residual bias is expected to have a stronger influence on model predictions in peripheral or poorly known habitats, where limited occurrence data can increase uncertainty and reduce the reliability of projected suitability. After data cleaning, the remaining number of occurrence points per species was as follows: *Q. robur* (*n* = 276), *Q. petraea* (*n* = 438), *Q. coccifera* (*n* = 390), *Q. suber* (*n* = 338), *Q. cerris* (*n* = 107), and *Q. pubescens* (*n* = 392). Occurrence points used in this study are given as a Supporting Information.

Calibration areas (M) were determined using *SDMToolbox* (Brown 2014) by incorporating species biology, dispersal potential, habitat continuity and topographic patterns, following the BAM framework (Soberon and Peterson 2005; Peterson et al. 2015). In this framework, M represents the set of areas a species could have accessed over relevant time periods, given its dispersal capacity and historical biogeographic constraints. Defining M in this way restricts background sampling to ecologically and geographically accessible environments, thereby avoiding overestimation of suitability in regions the species cannot realistically occupy. Due to the known artifacts, four bioclimatic variables (bio8, bio9, bio18, and bio19) were excluded from the bioclimatic variables set. Climate variables were then masked to the species‐specific M calibration areas prior to modeling. To reduce multicollinearity, we performed Pearson correlation analysis used the “*corrplot*” package (version 0.92; Wei and Simko 2021) in R (R Core Team 2022). Among pairs of highly correlated variables (*r* > 0.70), one was retained, whereas the others were excluded (Figures S1–S6). In cases where pairwise correlations exceeded 0.70, we retained the variable judged to be more ecologically relevant to species' physiology and known distributional limits (e.g., temperature seasonality or precipitation variability) and discarded its correlated counterpart. When multiple variables were ecologically suitable, we prioritized those with broader support in the literature or clearer interpretability for comparative modeling. After collinearity removal, the final set of predictors consisted of four different variables for each species (Table S5). Under this modeling framework, we assume temporal stationarity in species–climate relationships, whereby climatic niches inferred from present‐day occurrences are transferable to past and future climatic conditions. Although common in correlative ENM (Peterson et al. 2015), this assumption may not fully capture shifts in species' realized niches under novel climatic regimes.

Modeling was conducted using MaxEnt version 4.3.3, which operates with presence‐only data (Phillips et al. 2006). Each model was run with 10 replicates. Model performance was assessed using the area under the curve (AUC) metric. As sufficient records were available for all species, default feature classes were used. Although we tested various regularization multipliers, they produced negligible differences in AUC values; thus, the default value of 1 was retained across all models. In addition to AUC, model performance was further evaluated using partial receiver operating characteristic (ROC) statistics, which better reflect predictive accuracy in presence‐only models (Peterson et al. 2008), by testing whether AUC‐ratio values were significantly greater than 1 using 1000 bootstrap iterations.

To convert continuous suitability outputs into binary presence–absence maps, we applied the sensitivity‐specificity equality threshold. This threshold is defined as the value at which sensitivity and specificity are equal, thereby minimizing both false positives and false negatives and maximizing overall predictive accuracy. In our models, the threshold ranged from 0.5 to 0.6 across species, with slight variation depending on species' distributions and ecological characteristics. Raster calculations and final visualizations of predicted distributions were conducted in ArcGIS 10.6.1 (ESRI 2010).

To complement visual inspection of ENM projections, we quantified distributional shifts for each species across past (LGM vs. Present) and future (Present vs. SSP1‐2.6, Present vs. SSP5‐8.5) scenarios. From binary presence–absence maps, we derived latitudinal centroid shifts, southern and northern range limit shifts, normalized area changes relative to the present distribution, and changes in north–south range extent. Metrics were extracted from georeferenced raster outputs in ArcGIS 10.6.1 (ESRI 2010) and cross‐checked using custom Python scripts (Python v3.x) employing the packages “*NumPy*” (Harris et al. 2020), “*pandas*” (McKinney 2010), and “*matplotlib*” (Hunter 2007). These values were calculated separately for each species and scenario, enabling quantitative comparison among Mediterranean, temperate, and transition‐zone oaks.

## 3 Results

### 3.1 Model Performance and Current Distribution Patterns

**FIGURE 1:**
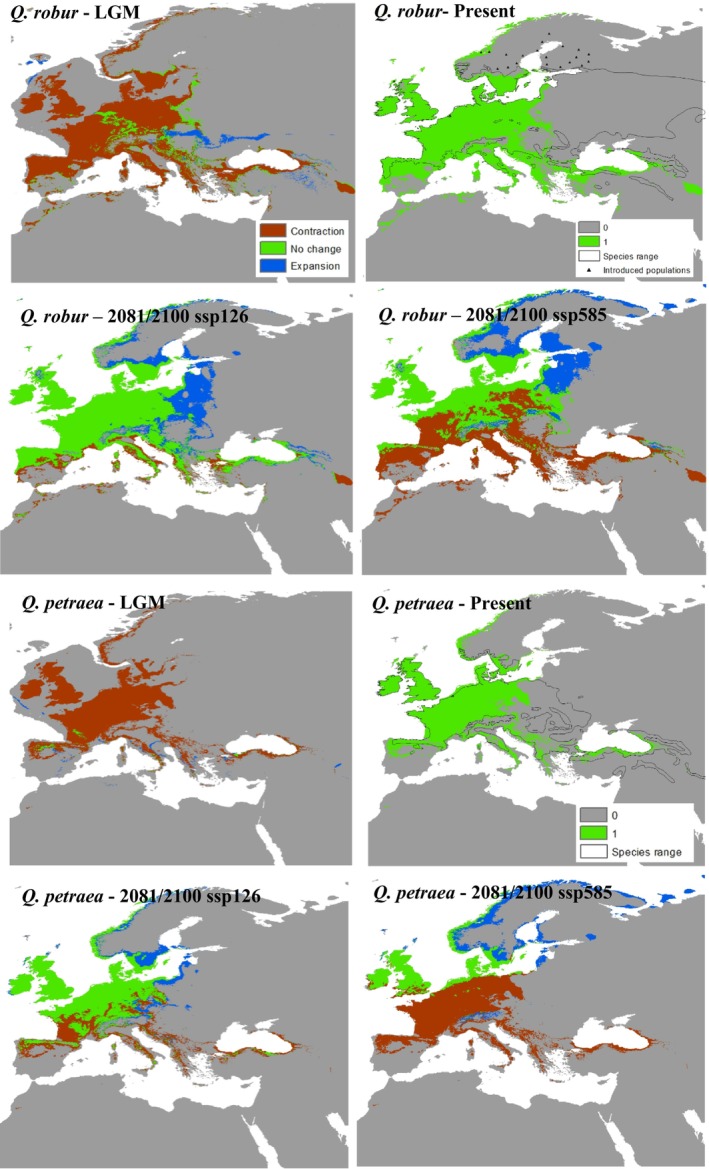
Modeled distributions of temperate‐zone species ( *Quercus robur* and *Quercus petraea* ) under climatic conditions of the Last Glacial Maximum (~22,000 years before present), the Present, and future projections (2081‐2100) under SSP1‐2.6 and SSP5‐8.5 scenarios.

**FIGURE 2:**
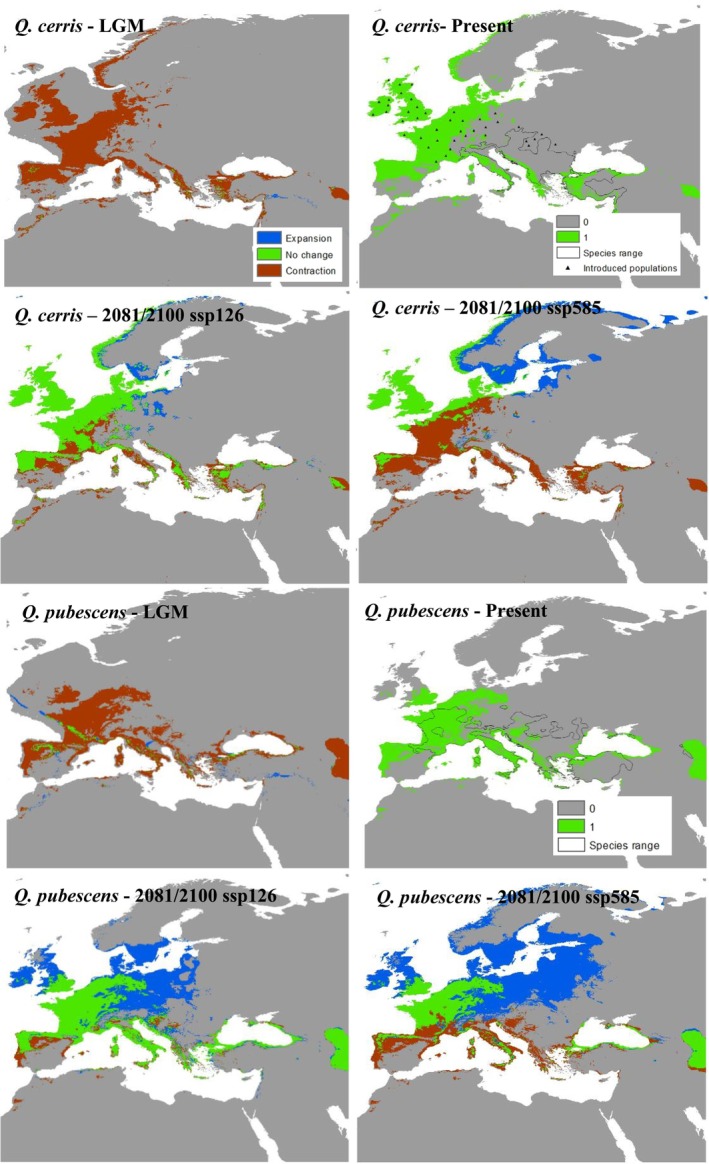
Modeled distributions of transition‐zone species ( *Quercus cerris* and *Quercus pubescens*) under climatic conditions of the Last Glacial Maximum (~22,000 years before present), the Present, and future projections (2081‐2100) under SSP1‐2.6 and SSP5‐8.5 scenarios.

**FIGURE 3:**
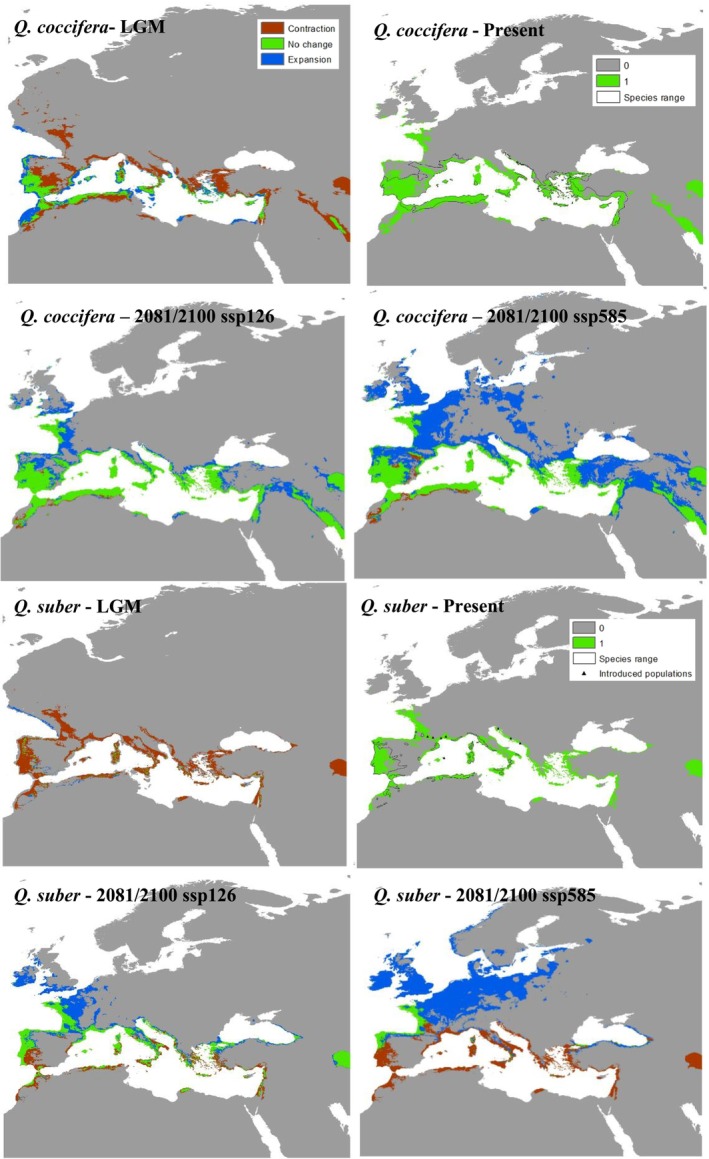
Modeled distributions of Mediterranean‐zone species (*Quercus coccifera* and *Quercus suber* ) under climatic conditions of the Last Glacial Maximum (~22,000 years before present), the Present, and future projections (2081‐2100) under SSP1‐2.6 and SSP5‐8.5 scenarios.

Ecological niche modeling projections generally showed strong concordance with the known current distributions of all study species (Figures 1, 2, 3), as confirmed by occurrence data from EUFORGEN (Caudullo et al. 2017) and GBIF (https://www.gbif.org/). However, the models underestimated eastern parts of the ranges of *Q. petraea* and *Q. robur* . This may reflect the scarcity of occurrence records from these regions, but the persistence of these species there also suggests the importance of additional factors beyond climate, such as local adaptation, historical dispersal routes, biotic interactions, or microclimatic conditions. Therefore, climate‐only projections should be interpreted cautiously, particularly when extrapolated beyond current environmental conditions. By excluding soil properties, biotic interactions, and anthropogenic filters, such models may overestimate the robustness of predicted distributions. Because our models rely solely on climatic variables, such processes could not be incorporated. Despite this limitation, our projections were broadly consistent with maximum habitat suitability maps provided by the European Atlas of Tree Species (San‐Miguel‐Ayanz et al. 2016). The models performed robustly across all species and time periods, with mean AUC values exceeding 0.75 (Table S3), indicating a strong predictive capacity relative to null expectations (Phillips and Dudík 2008). Partial ROC ratios were significantly greater than 1.0 (*p* < 0.001) for all species studied (Table S3), indicating that model predictions performed better than random expectations. Additionally, the bioclimatic response curves (Figures S7–S12) highlight the relative influence of individual variables on MaxEnt outputs and illustrate how changes in environmental parameters affect predicted suitability. Taken together, these complementary metrics indicate that the models provided robust and reliable predictions of species' climatic suitability.

Because the calibration areas were broadly defined to reflect known distributions, some climatically suitable areas outside current range were identified, for example, northwestern Europe and Britain for *Q. pubescens* (Figure 2), the Iberian Peninsula for *Q. cerris* (Figure 2), and the Aegean and southern Anatolian coasts for *Q. suber* (Figure 3). These patterns reflect the potential climatic equilibrium and reveal the ecological niches of the study species (Nogues‐Bravo 2009). However, recognizing the constraints posed by dispersal limitations, geographical barriers, and potential biotic interactions (e.g., competition and facilitation), we interpreted these projections cautiously to avoid overestimating the geographical range of species under different climatic scenarios. At the same time, we acknowledge that apparent absences in the current ranges may also reflect model uncertainty or incomplete predictor coverage, and ENMs alone cannot disentangle these possibilities. As with all equilibrium‐based niche models, our results indicate potential climatically suitable areas rather than realized distributions and should be considered as upper estimates of species' potential ranges.

Current climate models effectively identified optimal climatic conditions for each species and enabled projections across different time frames. Temperature‐related variables contributed most to the models for both temperate and transition‐zone species, accounting for over 80% of the explanatory power. Among these, temperature variability indices such as bio4 (temperature seasonality) and bio7 (temperature annual range) had the highest impact. In contrast, the distribution of Mediterranean species was influenced by both temperature‐ and precipitation‐related variables, although variables such as bio6 (minimum temperature of the coldest month) appeared to impose significant constraint on their distributions.

### 3.2 LGM Projections

Under LGM conditions, temperate and transition‐zone species exhibited similar range contraction patterns toward climatically suitable areas within their historical ranges (Figures 1, 2). Notable refugial regions included the Caucasus, coastal areas of the Black Sea, interior Aegean coasts and the Taurus Mountains in Anatolia, the Balkans, Italy, southern France, and the southern Pyrenees. In contrast, Mediterranean species maintained more stable distributions within the Mediterranean basin and showed either minimal change or expansion relative to temperate and transition‐zone species (Figure 3). Quantitative metrics confirmed these patterns, with temperate and transition‐zone species exhibiting marked contractions in suitable area and reductions in north–south range extent, whereas Mediterranean species showed comparatively stable or slightly expanded areas with limited centroid shifts (Tables 1 and S4).

**TABLE 1:** Summary of range‐shift metrics for the studied *Quercus* species across past (LGM vs. Present) and future climate scenarios (Present vs. SSP1‐2.6 and Present vs. SSP5‐8.5). Reported values include latitudinal centroid shift (ΔLat_cent, °N), southern and northern range limit shifts (ΔSouth and ΔNorth, °N), and normalized area change (% relative to Present). Positive values indicate northward centroid movement, poleward range shifts, or area expansion, whereas negative values indicate southward movement, equatorward shifts, or area contraction. Raw values for each species and scenario are provided in Table S4.

Scenario	ΔLat_cent (°N)	ΔSouth (°N)	ΔNorth (°N)	ΔArea (%)
**Temperate species**				
*Quercus robur*				
LGM	−11.27	−21.30	−4.45	−61.2
SSP1‐2.6	0.73	−0.23	−0.13	15.8
SSP5‐8.5	3.78	5.26	2.18	−11.9
*Quercus petraea*				
LGM	−2.57	3.48	−9.74	−97.9
SSP1‐2.6	1.82	5.21	1.16	−11.6
SSP5‐8.5	4.77	7.53	2.58	−45.9
**Transition‐zone species**				
*Quercus cerris*				
LGM	−2.89	2.81	−12.86	−99.4
SSP1‐2.6	1.58	0.28	1.12	−13.2
SSP5‐8.5	4.91	−2.24	2.23	−25.6
*Quercus pubescens*				
LGM	−1.22	0.76	−1.61	−95.0
SSP1‐2.6	1.43	2.31	4.95	75.7
SSP5‐8.5	4.26	4.39	9.47	105.7
**Mediterranean species**				
*Quercus coccifera*				
LGM	−0.90	−0.23	−2.14	−45.7
SSP1‐2.6	0.65	−0.13	3.22	63.2
SSP5‐8.5	1.89	−0.56	9.97	214.7
*Quercus suber*				
LGM	0.74	6.16	0.09	−95.6
SSP1‐2.6	1.89	5.16	4.09	34.4
SSP5‐8.5	5.57	9.43	11.43	148.5

### 3.3 Future Projections

Future projections under both climate scenarios suggest that temperate and transition‐zone species will continue to exhibit similar patterns, with range shifts toward the northern parts of the western Palearctic (Figures 1, 2). However, transition‐zone species appear unlikely to shift, maintaining a larger portion of their current distributions and even showing some expansion under future conditions. In contrast, temperate species are projected to experience substantial range contractions, especially under the pessimistic scenario, where they face near extinction within their present‐day ranges. Conversely, Mediterranean species are expected to expand their distributions into more temperate regions of the Palearctic, under both climate scenarios, with the realized extent of this expansion likely to be strongly mediated by land‐use legacies and disturbance regimes, particularly fire, as well as by dispersal limitation or facilitation through associated species and landscape connectivity, though slight southern contractions may occur. (Figure 3). The quantitative metrics highlight these contrasts (Tables 1 and S4): under SSP5‐8.5, temperate species ( *Q. robur* and *Q. petraea* ) exhibited pronounced northward centroid shifts (up to several degrees latitude) and consistent retreats of their southern limits, coupled with modest net area changes. Transition‐zone species (*Q*. *pubescens* and *Q. cerris* ) closely mirrored this temperate pattern in centroid and southern range shifts. In contrast, Mediterranean species (*Q*. *coccifera* and *Q. suber* ) showed more limited centroid shifts but substantially greater area expansion, driven mainly by the emergence of suitable climatic areas in northern regions (Tables 1 and S4).

## 4 Discussion

The high concordance between current model projections and known species distributions validates the use of our models for reconstructing past ranges and forecasting future distributions. Our findings show that oak species occupying different climatic zones within the western Palearctic exhibit distinct responses to climatic change. As hypothesized, Mediterranean and temperate oaks differed significantly in their range shifts. Quantitative metrics confirmed these contrasts: under SSP5‐8.5, temperate species ( *Q. robur* and *Q. petraea* ) displayed pronounced northward centroid shifts (up to ~5° latitude) and consistent contractions of their southern limits, accompanied by modest or negative area changes. In contrast, Mediterranean species (*Q*. *coccifera* and *Q. suber* ) exhibited more limited centroid displacement (< 2° latitude) but substantial area expansion (up to +200% relative to Present), driven largely by the emergence of suitable habitats in northern regions. Transition‐zone species ( *Q. cerris* and *Q*. *pubescens*) aligned more closely with the temperate group in terms of centroid shifts and southern range contractions, though *Q*. *pubescens* showed partial expansion under moderate scenarios. This pattern may be explained by a combination of ecological, evolutionary, and methodological factors. Ecologically, it may reflect local adaptation to specific microclimatic or topographic conditions, or evolutionary affinities that align these species more closely with one climatic group than the other. From an evolutionary perspective, the two transition‐zone species ( *Q. cerris* and *Q. pubescens*) belong to different oak sections (section *Cerris* and section *Quercus*), and neither to the *Ilex* section, which appears to be more strictly adapted to Mediterranean‐type climates. This phylogenetic background could partly explain why the observed responses for both transition‐zone species more closely resemble temperate patterns. Besides, in some cases ecotonal regions may also be dominated by one set of climatic constraints, such as seasonality or extreme temperature tolerance, resulting in patterns resembling one end of the climatic spectrum. Methodologically, because calibration areas were defined broadly, partial overlap with Mediterranean or temperate ranges could reinforce a stronger climatic signal and contribute to niche similarity, reducing the likelihood of detecting an “intermediate” pattern. In addition, some regional mismatches were observed, particularly the underestimation of eastern range limits of *Q. petraea* and *Q. robur* , likely reflecting both uneven occurrence data coverage and the influence of non‐climatic factors, such as local adaptation, dispersal history, and microclimatic heterogeneity. Moreover, although occurrence records were filtered to reduce spatial bias, underrepresentation of the species' full range in available databases may also have influenced the projected patterns. Taken together, these results highlight the ecological distinctness of Mediterranean oaks, whose climatic suitability may expand through northward range shifts rather than contract, and indicate that western Palearctic oaks distributed across Mediterranean, temperate, and transition zones will be differentially affected by future climate change.

Temperate (*Q. robur* and *Q. petraea*) and transition‐zone species (*Q. cerris* and *Q. pubescens*) contracted their ranges during the LGM toward climatically suitable refugia in southern Europe and Anatolia, consistent with the expansion–contradiction model and previous studies on temperate taxa (Bennett et al. 1991; Hewitt 1996; Bennett and Provan 2008; Provan and Bennett 2008; Médail and Diadema 2009). Besides the well‐established glacial refugia in the Iberian Peninsula, Italy, the Balkans, and Anatolia for temperate oak species in southern Europe (Bennett et al. 1991; Taberlet et al. 1998; Brewer et al. 2002; Bagnoli et al. 2016; Ülker et al. 2018), our results emphasize Anatolia's importance as a plausible refugium for transition‐zone oak species. Moreover, the Black Sea coast, southern France, and the Caucasus may represent extra‐Mediterranean refugia (Schmitt and Varga 2012; Perktaş et al. 2015). Due to moisture limitations, temperate oaks likely persisted in fragmented and spatially restricted forests during the LGM. Notably, the negative effects of drought are relatively less pronounced for temperate oaks growing in mesic habitats compared with those in drier environments (Bose et al. 2024). The strong similarity of transition‐zone species to temperate, rather than intermediate, patterns therefore represents an important and somewhat unexpected outcome of our analyses. Although this may reflect shared evolutionary histories, conserved climatic niches, or asymmetric ecological filtering at biome boundaries, disentangling these mechanisms requires targeted analytical approaches beyond the scope of this study. Future work could explicitly test these hypotheses using discriminant analyses to assess niche differentiation among species groups, as well as phylogenetic signal tests to evaluate the extent to which shared ancestry constrains observed response patterns. Their postglacial northward expansion, particularly during the Mid‐Holocene (~6000 BP), was likely accelerated by human activity (Turner et al. 2008), providing a potential explanation for the Reid Paradox, the discrepancy between rapid postglacial range expansion inferred from fossil records and biologically expected dispersal rates (Skellam 1951).

In contrast, Mediterranean species ( *Q. suber* , *Q*. *coccifera*) experienced less contraction and retained more stable distributions during the LGM. Biodiversity hotspots identified in the Mediterranean basin (Médail and Diadema 2009) coincided with modeled refugia of these species. Our findings align with paleoecological evidence indicating long‐term persistence of oak‐dominated vegetation in the region. Macrofossil records indicate that evergreen oaks have existed in the Eastern Mediterranean since the Miocene (Vitelli et al. 2017), with Anatolia and the Middle East functioning as both refugia and centers of diversification for the *Ilex* section. These findings support the multiple refugia hypothesis (de López Heredia et al. 2007) rather than the classic view of limited southern refugia. The restricted modern distribution of evergreen oaks to coastal areas of the Mediterranean may be influenced not only by climate but also by low competitive ability, fire regimes, herbivory, and pathogens (Carrión 2002; Turner et al. 2008). More generally, such non‐climatic drivers are also likely to affect the ranges of other oak species across the western Palearctic.

Future climate projections (SSP1‐2.6 and SSP5‐8.5) suggest that temperate and transition‐zone species will lose their southern populations but expand into northern latitudes and higher elevations, particularly in Scandinavia. Transition‐zone species appear more resistant, maintaining broader southern populations than temperate species. However, in the pessimistic scenario, temperate oaks, *Q. petraea* and *Q. robur* , two of the most ecologically and economically important European forest species (Cottrell et al. 2002), are projected to face substantial range reductions. Mediterranean species, in contrast, are projected to expand under both climate scenarios, although slight southern contractions are anticipated. Indeed, even under the pessimistic scenario in which warming is expected to cause the mid‐latitudes of Europe to gradually acquire Mediterranean climate characteristics (Pörtner et al. 2022), *Q. coccifera* is likely to extend its range into central Europe, potentially benefiting from the northward shift of Mediterranean‐like conditions. *Q. suber* , however, may lose some southern populations while expanding northeastward. Its more limited adaptability compared with *Q*. *coccifera* also highlights species‐specific vulnerabilities to climate change. Furthermore, the emergence of novel fire regimes in temperate Europe (Grünig et al. 2023; Sayedi et al. 2024) may further facilitate the northward expansion of Mediterranean oaks, given their strong resprouting ability even after intense crown fires (Tavşanoğlu and Pausas 2018). However, physiological responses of species to climatic stressors are often more complex than ecological niche models can account for; for example, whereas Mediterranean oaks can tolerate isolated drought years, their survivorship declines markedly under repeated drought events (Bose et al. 2024). Our projections rely on equilibrium‐based niche models derived from present‐day occurrence–climate relationships. They do not explicitly incorporate other important factors, such as adaptive capacity, genetic diversity, or biotic interactions, all of which can strongly influence species' responses to climate change. For instance, population‐level variation in drought tolerance (Ghouil et al. 2020) can be as consequential as interspecific differences (Malyshev et al. 2016), potentially altering range expansion–contradiction dynamics. Therefore, our results should be interpreted as estimates of potential climatic niches, rather than definitive predictions of realized future ranges.

The contrasting responses of oak species across climatic zones reflect underlying variation in the climatic variables shaping their distributions. Temperate and transition‐zone species appear more sensitive to climate change, whereas Mediterranean species demonstrate greater tolerance or adaptation to climatic variability. These patterns are consistent with the well‐established relationship between ecological niches and spatial characteristics of species under changing climates (Thuiller et al. 2005). However, although temperature and precipitation are significant drivers, other non‐climatic processes such as biotic interactions, topographic heterogeneity, soil conditions, and competition also play crucial roles in shaping distributions, particularly in transition zones where species are subject to multiple ecological pressures. Beyond climatic constraints, a range of biotic interactions may further modify the realized responses of oak species to climate change, particularly in Mediterranean ecosystems. Competitive interactions with co‐occurring tree and shrub species can influence regeneration success and local abundance, thereby affecting range filling under changing environmental conditions. In addition, pathogens and insect herbivores, whose impacts are often amplified under drought stress, may further constrain population persistence or limit expansion at distribution margins (Bose et al. 2024). Mutualistic interactions, particularly with ectomycorrhizal fungi, have been shown to alter physiological and biochemical responses of Mediterranean oaks to drought stress and may therefore influence regeneration and demographic performance under increasing aridity (Sebastiana et al. 2018, 2019). Together, these biotic processes interact with climate and disturbance regimes, underscoring the limits of climate‐only projections. Further research that explicitly incorporates these additional processes will help refine our understanding of realized distributions and species sensitivities. In the context of the European climate, structured by a dominant temperature gradient and a secondary precipitation gradient (Thuiller et al. 2005; Peel et al. 2007), the niche positions of species along these axes help explain their differential responses to past and projected climatic shifts. These findings highlight the complexity of predicting species' range shifts and underscore the need for a more holistic approach that integrates both climatic and non‐climatic determinants. By combining paleoclimatic reconstructions with future climate projections, our study enhances understanding of how late Quaternary climatic dynamics have shaped present‐day oak diversity in the western Palearctic and sheds light on the biogeographical patterns and climate sensitivities of temperate and Mediterranean oaks.

## 5 Conclusions

Our study demonstrates that oak species occupying different climatic zones in the western Palearctic exhibit distinct distributional responses to past and future climate change. Although temperate and transition‐zone oaks contracted their ranges during the LGM and are projected to face significant habitat loss under future warming, especially under pessimistic scenarios, Mediterranean oaks have shown greater historical stability and potential for range expansion. The differential responses among species may reflect underlying differences in climatic tolerance, ecological traits, and evolutionary histories. Nevertheless, projections derived from climate‐only ecological niche models should be interpreted with caution. Unforecasted changes may arise from biotic interactions, evolving management practices, or the emergence of novel climatic conditions beyond the range of contemporary analogs. Accordingly, ENM outputs should be viewed as testable hypotheses rather than definitive forecasts, requiring validation through targeted field observations and long‐term monitoring. Embedding these projections within adaptive management frameworks will be essential to iteratively assess model expectations, detect unexpected responses, and adjust conservation actions accordingly. Our results emphasize the need to incorporate regional climatic context and species‐specific traits into future projections, advocating for climate‐zone‐specific conservation strategies. For Mediterranean species, priorities could include conserving drought‐tolerant populations, enhancing habitat connectivity, and integrating adaptive fire‐management practices. For temperate species, strategies might focus on maintaining corridors that facilitate latitudinal or altitudinal range shifts and monitoring for emerging pest and pathogen threats. For transition‐zone species, management could aim to preserve populations with tolerance to both drought and cold stress while safeguarding genetic diversity to support adaptive potential under uncertain climatic futures. Such tailored approaches will be essential to enhance the resilience of European oak forests under ongoing and future climate change.

## Author Contributions

**Elif Deniz Ülker:** data curation (lead), formal analysis (lead), investigation (lead), methodology (equal), resources (lead), software (lead), validation (equal), visualization (lead), writing – original draft (lead), writing – review and editing (equal). **Çağatay Tavşanoğlu:** conceptualization (lead), formal analysis (supporting), funding acquisition (lead), investigation (supporting), methodology (equal), project administration (lead), resources (supporting), software (supporting), supervision (lead), validation (equal), writing – review and editing (equal).

## Funding

This work was supported by the Hacettepe Üniversitesi (FDK‐2018‐17218).

## Conflicts of Interest

The authors declare no conflicts of interest.

## Supporting information


**Data S1:** ece373055‐sup‐0001‐Supinfo.zip.

## Data Availability

Raw occurrence data used in the study are provided in the Supporting Information.
