# Supplementary material for: Oaks and Climate Change: Contrasting Range Responses of Mediterranean and Temperate Quercus Species in the Western Palearctic
Source: Ecol Evol. 2026 Feb 6;16(2):e73055. doi: 10.1002/ece3.73055 (PMC12880897; doi:10.1002/ece3.73055)
Supplement: Supplementary file 1 — Data S1: ece373055‐sup‐0001‐Supinfo.zip. [file ECE3-16-e73055-s001.zip › Supporting_Information.docx]

**SUPPORTING INFORMATION**

**Oaks and Climate Change: Contrasting Range Responses of Mediterranean and Temperate *Quercus* Species in the Western Palearctic**

**Table S1.** The links for specific GBIF datasets downloaded for each *Quercus* species included in the study.

| **Species** | **Download link** |
| --- | --- |
| *Q. robur* | https://doi.org/10.15468/dl.53ahkx |
| *Q. petraea* | https://doi.org/10.15468/dl.kqnxbx |
| *Q. coccifera* | https://doi.org/10.15468/dl.fcwra8 |
| *Q. suber* | https://doi.org/10.15468/dl.mfcee4 |
| *Q. cerris* | https://doi.org/10.15468/dl.dnns4w |
| *Q. pubescens* | https://doi.org/10.15468/dl.kumr2u |

**Table S2.** Bioclimatic variables used in ecological niche models (the WorldClim database version 2.1).

| **Bioclimatic Variable** | **Definition** |
| --- | --- |
| BIO1 | Annual mean temperature |
| BIO2 | Mean diurnal range (max temp./min temp.)(monthly average) |
| BIO3 | Isothermality (BIO1/BIO7) × 100 |
| BIO4 | Temperature seasonality (standard deviation × 100) |
| BIO5 | Maximum temperature of the warmest month |
| BIO6 | Minimum temperature of the coldest month |
| BIO7 | Temperature annual range (BIO5-BIO6) |
| BIO8 | Mean temperature of wettest quarter |
| BIO9 | Mean temperature of driest quarter |
| BIO10 | Mean temperature of warmest quarter |
| BIO11 | Mean temperature of driest quarter |
| BIO12 | Annual precipitation |
| BIO13 | Precipitation of the wettest month |
| BIO14 | Precipitation of the driest month |
| BIO15 | Precipitation seasonality (Coefficient of Variation) |
| BIO16 | Precipitation of wettest quarter |
| BIO17 | Precipitation of driest quarter |
| BIO18 | Precipitation of warmest quarter |
| BIO19 | Precipitation of coldest quarter |

**Table S3.** Mean AUC and partial ROC values of the models for each species. P-values from partial ROC analysis testing whether AUC-Ratio values are significantly greater than 1.

| Species | AUC |  | Partial ROC Statistics | |
| --- | --- | --- | --- | --- |
|  |  |  | AUC-Ratio | P |
| *Q. robur* | 0.737 |  | 1.067 | < 0.001 |
| *Q. petraea* | 0.830 |  | 1.106 | < 0.001 |
| *Q. coccifera* | 0.817 |  | 1.314 | < 0.001 |
| *Q. suber* | 0.840 |  | 1.105 | < 0.001 |
| *Q. cerris* | 0.772 |  | 1.153 | < 0.001 |
| *Q. pubescens* | 0.789 |  | 1.045 | < 0.001 |

**Table S4.** Complete set of range-shift metrics for the studied *Quercus* species across past (LGM vs. Present) and future climate scenarios (Present vs. SSP1-2.6 and Present vs. SSP5-8.5). For each species and scenario, we report centroid coordinates (latitude and longitude, °), southern and northern range limits (°N), minimum and maximum longitudes (°E), and north-south and east-west extents (°).

| **Scenario** | **Lat_centr** | **Lon_centr** | **Lat_min** | **Lat_max** | **Lon_min** | **Lon_max** | **NS_width** | **EW_width** |
| --- | --- | --- | --- | --- | --- | --- | --- | --- |
| **Temperate species** | | | | | | | | |
| ***Quercus robur*** | | | | | | | | |
| LGM | 47.29 | 19.47 | 27.71 | 65.74 | -15.17 | 51.36 | 38.02 | 66.53 |
| Present | 58.57 | -3.63 | 49.01 | 70.18 | -15.62 | 18.1 | 21.18 | 33.72 |
| SSP1-2.6 | 59.3 | -2.21 | 48.78 | 70.06 | -15.64 | 19.28 | 21.28 | 34.92 |
| SSP5-8.5 | 62.35 | -0.64 | 54.27 | 72.37 | -14.39 | 22.41 | 18.1 | 36.8 |
| ***Quercus petraea*** | | | | | | | | |
| LGM | 56.12 | -2.77 | 52.31 | 59.55 | -13.81 | 18.53 | 7.24 | 32.33 |
| Present | 58.69 | -4.81 | 48.83 | 69.29 | -14.94 | 15.91 | 20.46 | 30.85 |
| SSP1-2.6 | 60.51 | -4.73 | 54.03 | 70.45 | -14.39 | 11.95 | 16.41 | 26.34 |
| SSP5-8.5 | 63.46 | -2.09 | 56.35 | 71.87 | -14.05 | 22.5 | 15.52 | 36.55 |
|  |  |  |  |  |  |  |  |  |
| **Transition-zone species** | | | | | | | | |
| ***Quercus cerris*** | | | | | | | | |
| LGM | 55.12 | 10.93 | 51.29 | 56.79 | -12.95 | 17.74 | 5.5 | 30.69 |
| Present | 58.01 | -5.51 | 48.48 | 69.65 | -15.78 | 18.3 | 21.17 | 34.07 |
| SSP1-2.6 | 59.6 | -5.62 | 48.77 | 70.77 | -15.88 | 19.04 | 22 | 34.93 |
| SSP5-8.5 | 62.92 | -2.01 | 46.24 | 71.88 | -17.96 | 22.63 | 25.65 | 40.59 |
| ***Quercus pubescens*** | | | | | | | | |
| LGM | 56 | -2.95 | 49.5 | 59.81 | -14.46 | 18.51 | 10.31 | 32.97 |
| Present | 57.21 | -1.9 | 48.74 | 61.42 | -15.34 | 19.62 | 12.68 | 34.97 |
| SSP1-2.6 | 58.65 | -1.79 | 51.05 | 66.36 | -14.57 | 19.42 | 15.31 | 33.99 |
| SSP5-8.5 | 61.47 | 0.8 | 53.13 | 70.89 | -14.14 | 19.56 | 17.76 | 33.69 |
|  |  |  |  |  |  |  |  |  |
| **Mediterranean species** | | | | | | | | |
| ***Quercus coccifera*** | | | | | | | | |
| LGM | 53.04 | -6.67 | 48.82 | 58.42 | -15.37 | 18.3 | 9.6 | 33.67 |
| Present | 53.94 | -1.71 | 49.04 | 60.56 | -15.26 | 19.05 | 11.52 | 34.31 |
| SSP1-2.6 | 54.59 | -1.41 | 48.91 | 63.78 | -15.89 | 19.23 | 14.87 | 35.12 |
| SSP5-8.5 | 55.83 | -0.23 | 48.48 | 70.53 | -15.65 | 19.79 | 22.05 | 35.44 |
| ***Quercus suber*** | | | | | | | | |
| LGM | 55.2 | -10.3 | 49.84 | 58.43 | -14.57 | 10.04 | 8.59 | 24.61 |
| Present | 54.46 | -2.86 | 43.68 | 58.34 | -18.5 | 19.5 | 14.66 | 38 |
| SSP1-2.6 | 56.36 | -4.31 | 48.84 | 62.43 | -15.51 | 19.58 | 13.59 | 35.09 |
| SSP5-8.5 | 60.03 | -4.19 | 53.11 | 69.77 | -13.86 | 14.83 | 16.66 | 28.69 |

**Table S5.** Final set of bioclimatic variables used in the models for each species.

| **Species** | **Set of Variables** |
| --- | --- |
| *Q. robur* | bio1, bio3, bio4, bio12 |
| *Q. petraea* | bio4, bio5, bio11, bio14 |
| *Q. coccifera* | bio3, bio6, bio16, bio17 |
| *Q. suber* | bio5, bio6, bio7, bio16 |
| *Q.cerris* | bio4, bio11, bio13, bio14 |
| *Q. pubescens* | bio5, bio7, bio11, bio14 |


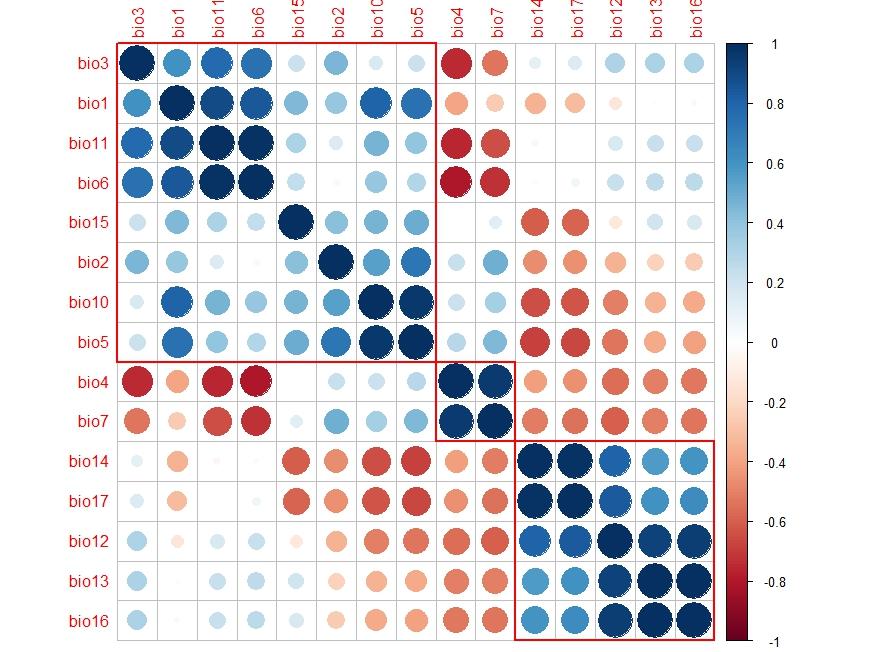


**Figure S1.** Correlation matrix showing associations among 15 bioclimatic variables for *Q. robur.*


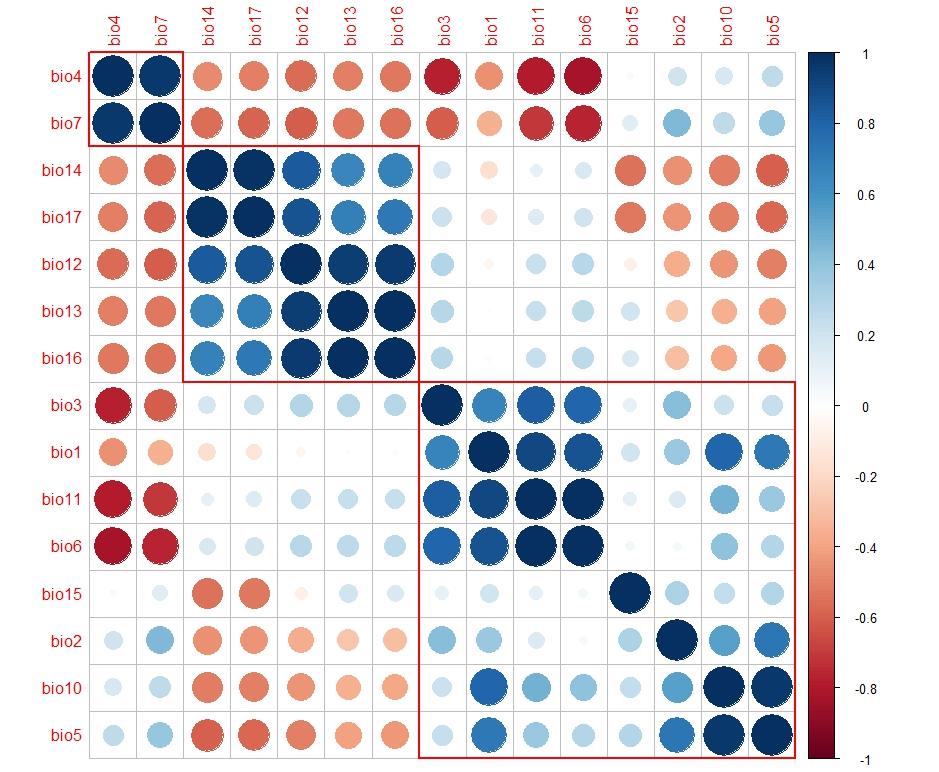


**Figure S2**. Correlation matrix showing associations among 15 bioclimatic variables for *Q. petraea.*


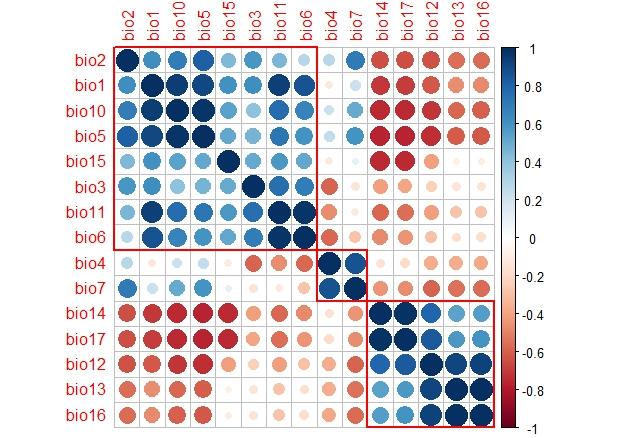


**Figure S3.** Correlation matrix showing associations among 15 bioclimatic variables for *Q. coccifera.*


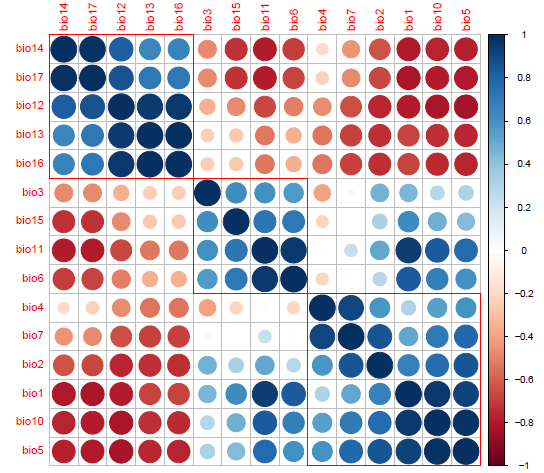


**Figure S4.** Correlation matrix showing associations among 15 bioclimatic variables for *Q. suber.*


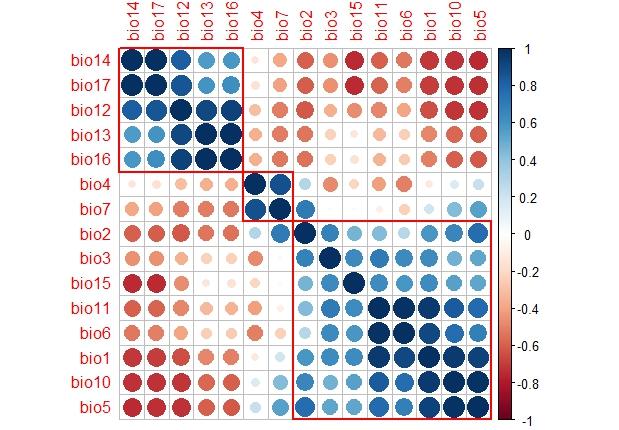


**Figure S5.** Correlation matrix showing associations among 15 bioclimatic variables for *Q. cerrris.*


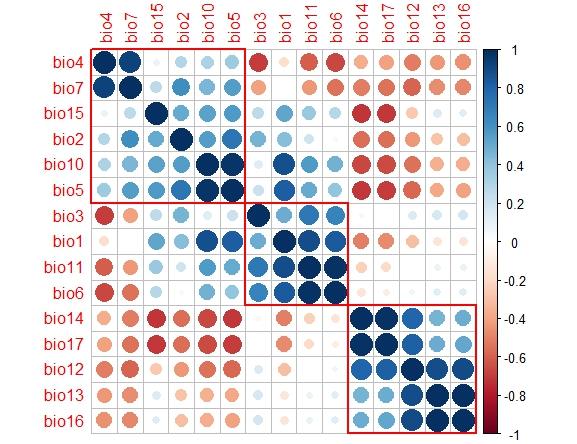


**Figure S6.** Correlation matrix showing associations among 15 bioclimatic variables for *Q. pubescens.*


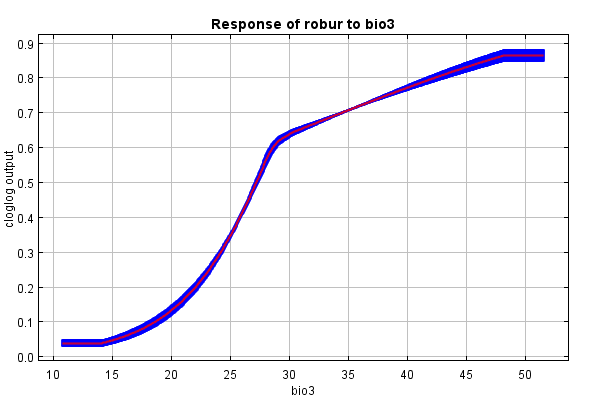

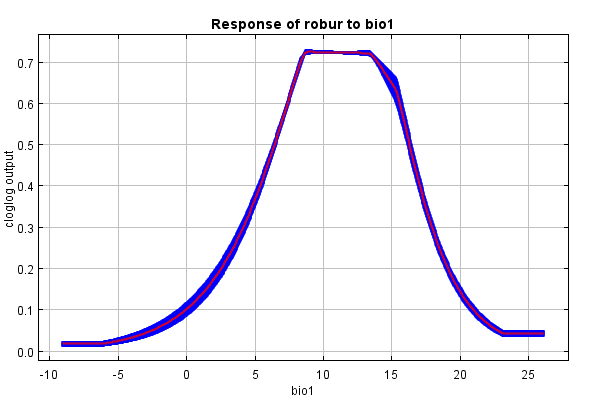


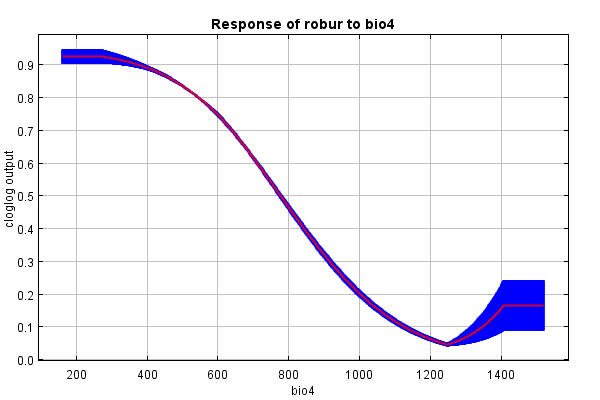

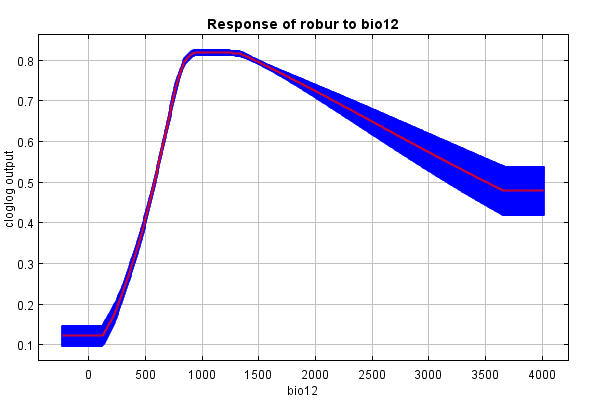


**Figure S7.** Response curves of the bioclimatic variables affected model projections for *Q. robur.*


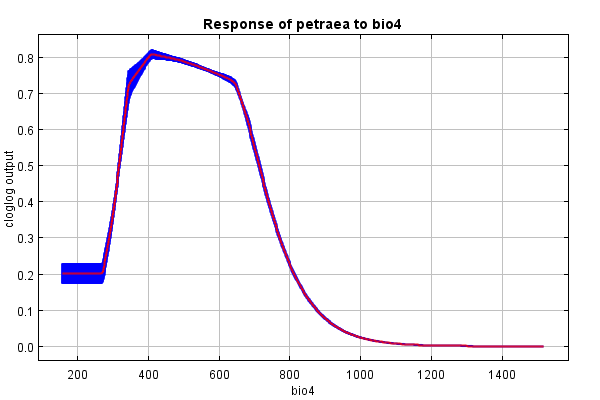

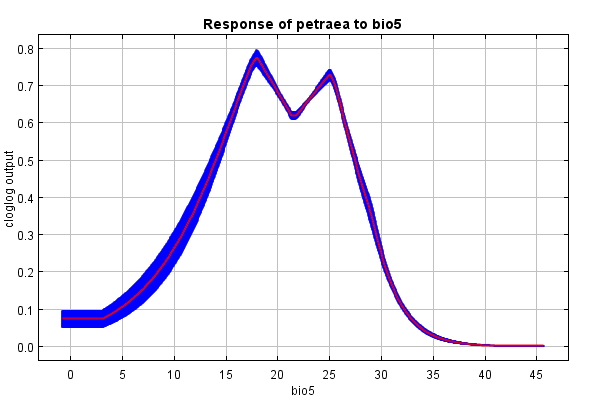


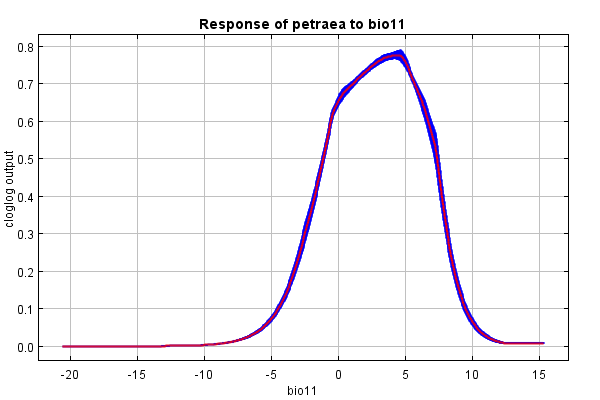

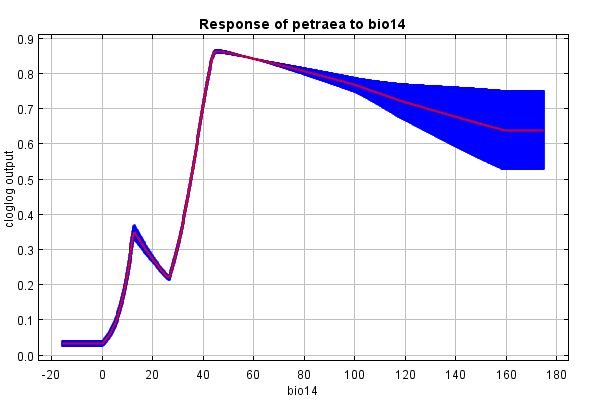


**Figure S8.** Response curves of the bioclimatic variables affected model projections for *Q. petraea.*


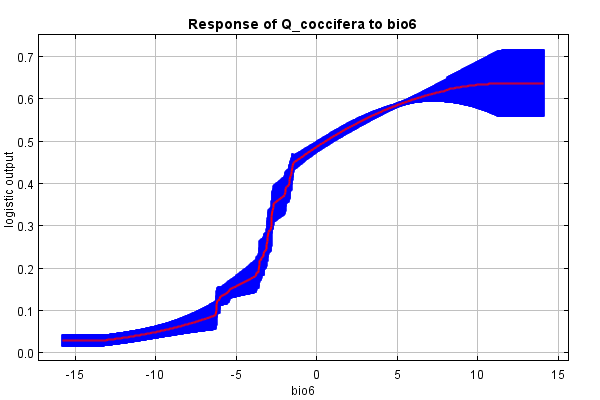

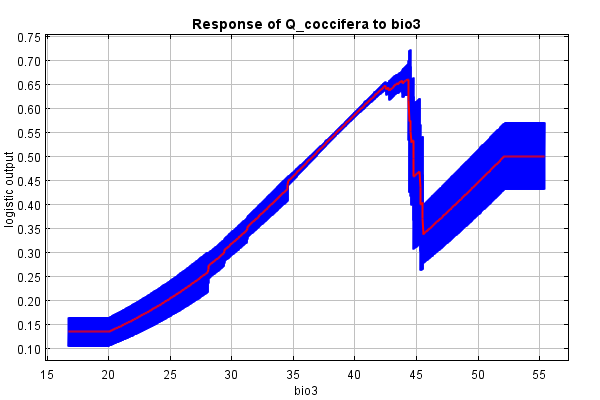


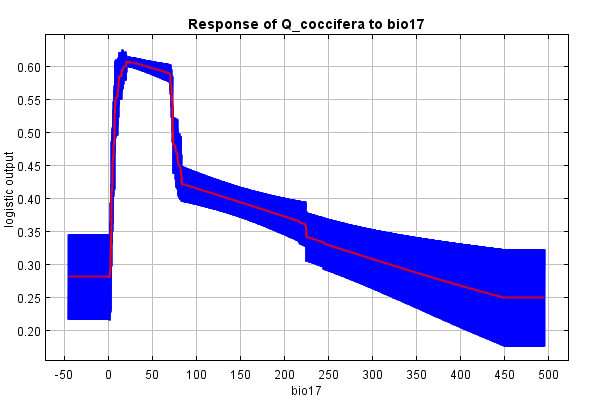

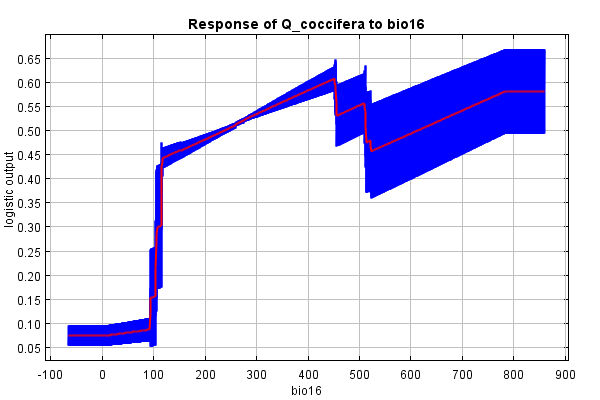


**Figure S9.** Response curves of the bioclimatic variables affected model projections for *Q. coccifera.*


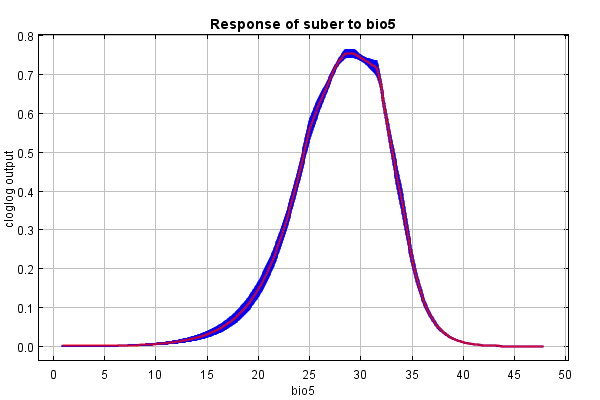

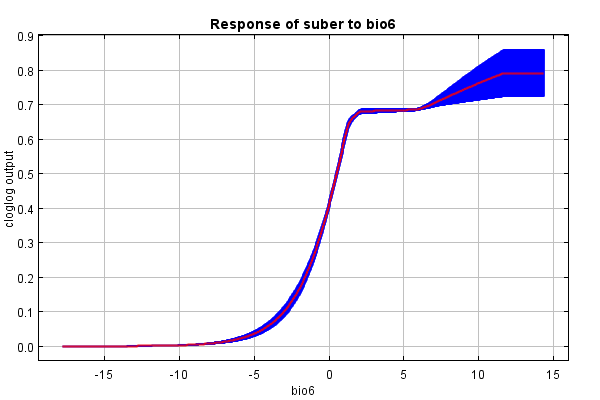


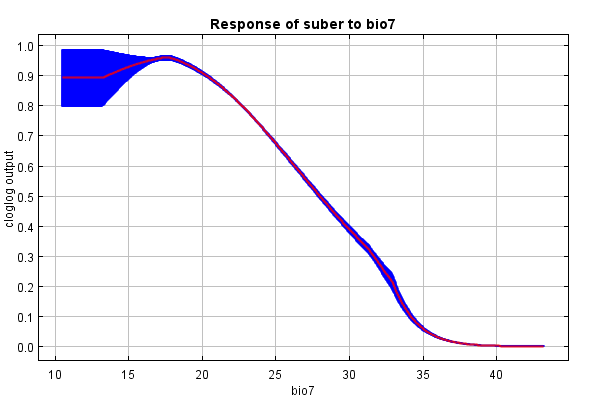

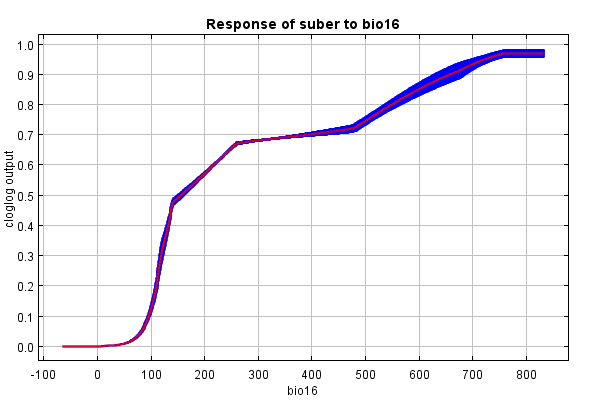


**Figure S10.** Response curves of the bioclimatic variables affected model projections for *Q. suber.*


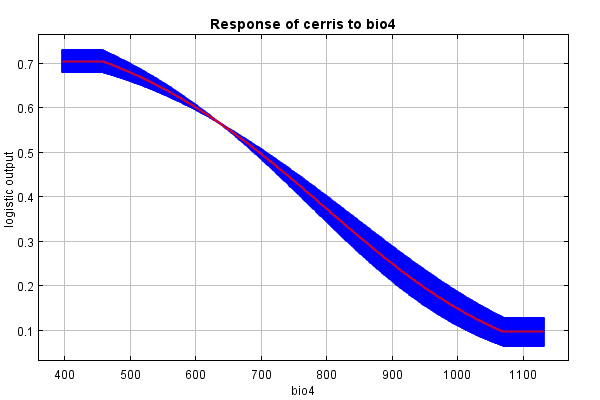

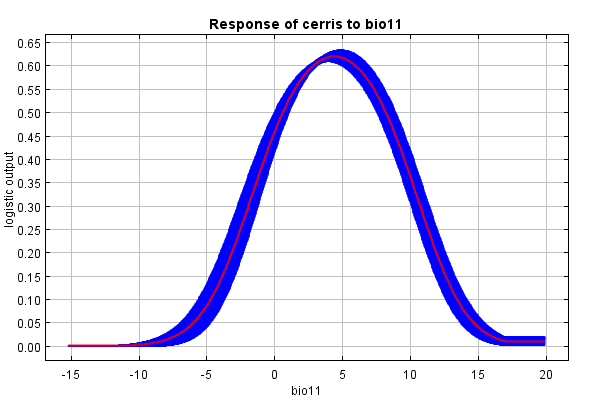


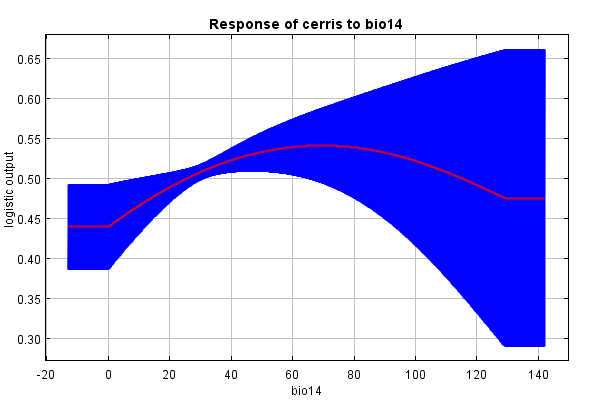

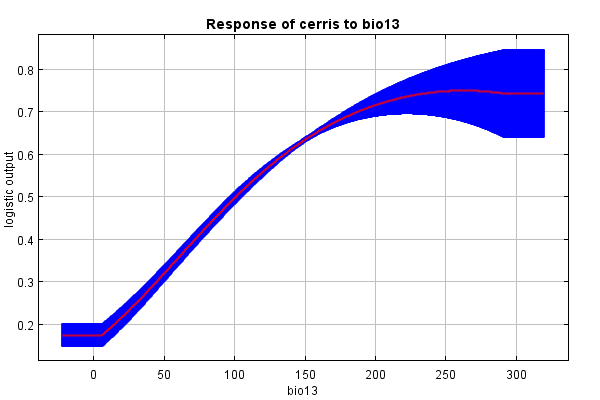


**Figure S11.** Response curves of the bioclimatic variables affected model projections for *Q. cerris.*


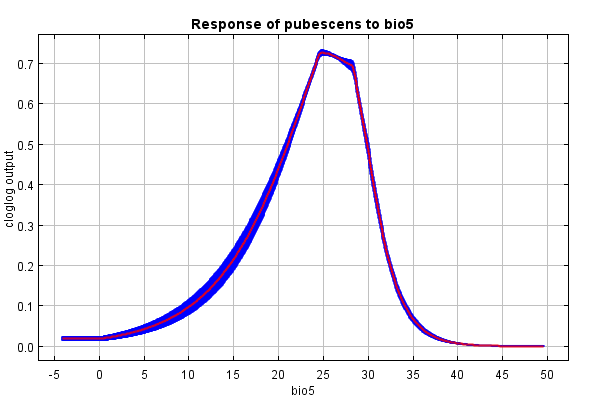

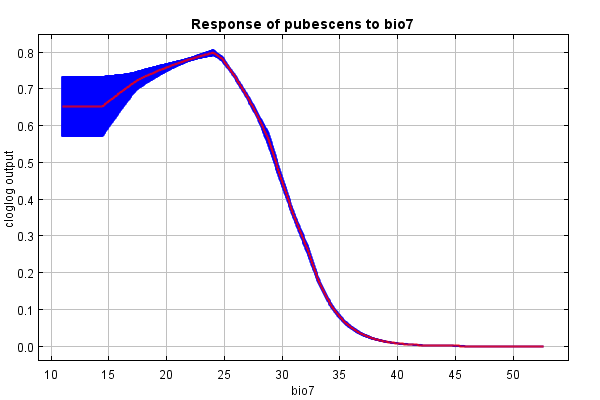


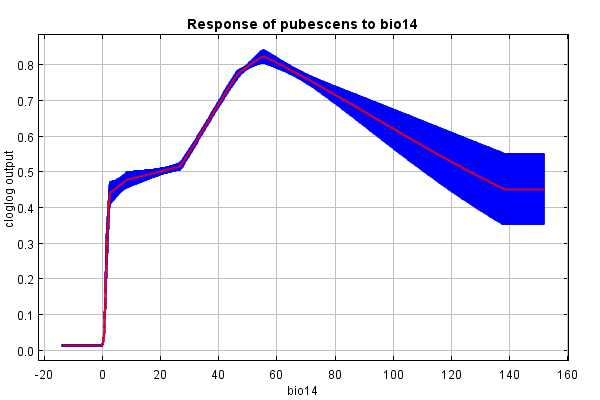

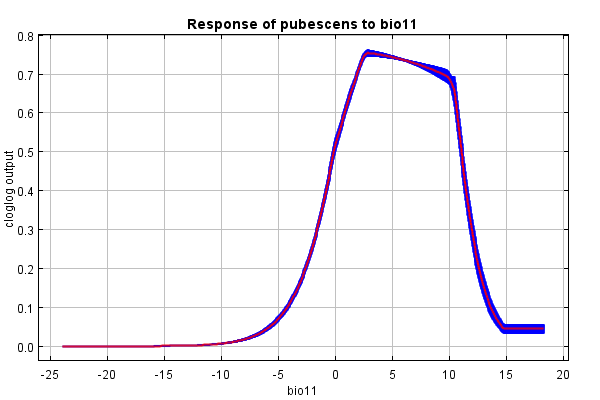


**Figure S12.** Response curves of the bioclimatic variables affected model projections for *Q. pubescens.*
